# The Effects of Extra-Team Goal Disclosure on Team Performance, Viability, and Satisfaction

**DOI:** 10.3389/fpsyg.2020.548842

**Published:** 2021-01-12

**Authors:** Esther Sackett, Gráinne M. Fitzsimons

**Affiliations:** ^1^Leavey School of Business, Santa Clara University, Santa Clara, CA, United States; ^2^Fuqua School of Business, Duke University, Durham, NC, United States

**Keywords:** teams, goal pursuit, team cognition, team processes, team performance

## Abstract

In addition to the team’s shared goals, team members also often hold goals unrelated to the team. Research about such goals, which we call “extra-team goals” (ETGs), has been limited. In the current research, we examine how awareness of a team member’s ETGs affects team outcomes. A laboratory experiment examines the effects of disclosure of different types of ETGs by one team member (target) on team performance, team viability, and team satisfaction while engaging in a brainstorming task. Our findings suggest that there are significant positive effects of ETG disclosure on team performance, team viability, and team satisfaction, and that these effects are mediated by perceptions of the target’s commitment to the team’s goal.

## Introduction

The pursuit of multiple goals is ubiquitous in everyday organizational life. Multiple times within the workday, people must make decisions about how to allocate their time, attention, and other resources toward the pursuit of work-related goals stemming from multiple projects, roles, and teams. In 1 day, a given team member may pursue: (a) team-level goals (e.g., for the team to submit a project by a certain date); (b) individual goals within the team context (e.g., to improve one’s skills on a team-related task); (c) relational goals within the team context (e.g., to impress a certain team member); and (d) goals that are not directly relevant to the team (e.g., to network with colleagues in a different department). Such goals are often interdependent, exerting both positive and negative influence on each other’s achievement. They may facilitate each other, as when a team member’s goal to network with a different department provides access to information that helps the team finish the project in a timely fashion. They may also conflict with each other, as when that same goal consumes time that would have been used for finishing the project.

The majority of research on team goal pursuit has focused on goal setting at the team level (O’Leary-Kelly et al., 1994; Nahrgang et al., 2013), and on relationships between individual and team level goals (DeShon et al., 2004; Chen and Kanfer, 2006; Crown, 2007; Chen et al., 2009). Building on this research, recent theory has highlighted the potential value in understanding other kinds of goals that affect team dynamics, including external goals directed toward outcomes outside of the team, or *extra-team goals*. Extra-team goals are an extremely diverse set of goals, including personal goals such as organizing a fundraiser, professional goals such as moving to a new division, and goals related to other teams within the organization (Fitzsimons et al., 2016). The defining feature of extra-team goals is that they do not directly relate to the team – they aren’t “about” the team’s goal outcomes but are instead “about” some other aspect of the individual’s organizational or personal life. Despite their definitional lack of direction toward the team, extra-team goals have powerful potential to affect the team’s outcomes. A team member’s pursuit of a goal to move to a new division, for example, may damage the team’s outcomes by conflicting with certain role assignments, or may facilitate the team’s outcomes by increasing exposure to new information. The current research does not seek to explore the effects of such goals, *per se*, but instead seeks to explore how team members’ *awareness* of each other’s extra-team goals affects team outcomes such as performance, satisfaction, and viability. We also explore perceptions of commitment and trust as mediating mechanisms of these relationships.

Recent research has theorized about the role of awareness of team members’ extra-team goals as being one potential key to increasing multiple goal coordination within the team (Fitzsimons et al., 2015, 2016; Pearsall and Venkataramani, 2015; Sackett, 2017). The extensive influence of other types of team cognition, such as team mental models (Converse et al., 1991; Mathieu et al., 2000; Lim and Klein, 2006; DeChurch and Mesmer-Magnus, 2010) and transactive memory systems (Moreland et al., 1996; Austin, 2003; Lewis, 2004) are well understood and robustly demonstrated in the literature. In the current research, we extend this body of work to explore the costs and benefits of awareness of others’ goals – specifically, those goals that relate to extra-team outcomes.

## The Potential Benefits and Costs of ETG Awareness

The extant literature on team cognition provides mixed evidence about the potential consequences of awareness of extra-team goals (ETGs hereafter) for team outcomes (Mathieu et al., 2000; Ethiraj and Levinthal, 2009; DeChurch and Mesmer-Magnus, 2010; Huber and Lewis, 2010; Pearsall and Venkataramani, 2015). While there are obvious and well-established benefits to being “on the same page” about the team’s shared goals, the benefits of being on the same page about ETGs are less clear. On the one hand, when ETGs are in conflict with the team’s interests, awareness of those goals could erode trust or cause interpersonal conflict, which is why individuals in such positions frequently withhold information about their goals (Wittenbaum et al., 2004). On the other hand, when ETGs are aligned with the team’s interests, awareness of those goals could have multiple different effects. For example, what if a team member has a goal to learn a new skill or be offered a promotion beyond the team and the team’s work provides a path to facilitate those goals? If the other team members become aware of those ETGs and their relationship to the team’s work, would it signal that member’s commitment to the team’s work, given their extra stakes? Or would it suggest that the member is using the team as a stepping stone, only to jump ship when their ETGs are attained?

To date, the consequences of ETG awareness for important team level outcomes such as team performance, satisfaction, and viability have not been empirically tested, with one notable exception. Pearsall and Venkataramani (2015) investigated how teams are able to overcome goal asymmetries by developing team goal mental models that included members’ personal goals, finding that teams who had high team identification and high team learning orientation developed planning processes and team goal mental models that enabled them to successfully pursue team goals. However, there is still very much about the interpersonal effects of becoming aware of members’ ETGs that is unknown – particularly how different types of ETGs may be perceived by other members.

### The Global Effects of ETG Disclosure on Performance, Viability, and Satisfaction

Because of the uncertainty regarding whether individuals will react to the ETGs of their fellow team members in a positive or a negative light, it is important to understand how these perceptions will impact not only team performance, but also individuals’ satisfaction with their team experience, and their perceptions of team viability – whether the team has the potential to thrive in the long term (Bell and Marentette, 2011) – all of which are important for the long term success of a team. While knowledge of team members’ ETGs could conceivably improve coordination leading to better performance, such knowledge could simultaneously erode trust or perceptions of commitment as a result of knowledge of competing motives. Or, knowledge of ETGs could help to resolve conflicts that may have been attributed to negative personal characteristics, which would aid satisfaction. These dynamics, in turn, could affect the long-term ability of a team to work together. Given that this is a novel question with no strong empirical precedent, we have chosen to pose a research question, rather than a specific hypothesis, about the direction of the global effects of ETG disclosure. While we have predictions, based on theory and empirical research, about some of the patterns we might expect to see regarding the relative effects of disclosing different *types* of ETGs (developed below), we hesitate to predict that disclosing all types of ETGs will have a unilaterally positive or negative effect on these outcomes. Furthermore, although there are a variety of ways in which a team member may ‘become aware’ of another member’s ETGs – for example, the goal holder could disclose the ETG, an ETG could be inferred, observed, or be divulged by someone else – we focus specifically on ETG disclosure as a path to ETG awareness, and the consequences of such disclosure. We will revisit the implications of focusing on disclosure, specifically, in our discussion.

Research Question 1: How does ETG disclosure affect team performance, viability, and satisfaction?

### The Effects of Different Types of ETG Disclosure on Performance, Viability, and Satisfaction

In contrast to the lack of evidence upon which to make a prediction regarding the global effects of disclosing ETGs, we drew upon a much more robust set of evidence regarding the effects of different types of goal content in order to make predictions about how different types of ETGs may affect team outcomes. Research from multiple literatures provides evidence that different types of goals have different effects on motivation and performance. For example, prior research on goal setting theory has demonstrated that setting goals with different types of content (i.e., those that emphasize learning outcomes vs. those that emphasize performance outcomes) does affect goal pursuit processes and outcomes in different ways in different contexts, both for individuals (Seijts and Latham, 2001, 2005) and for teams (Nahrgang et al., 2013). For example, Seijts and Latham (2005) found that in situations in which acquisition of new knowledge or skills is necessary, specific learning goals can be more beneficial for individuals than performance outcome goals (which are most useful in situations in which effort or persistence on a known task are needed). Nahrgang et al. (2013) found that, for teams, general learning goals (i.e., “learn what you can”), as opposed to specific learning goals or specific performance goals, can be most beneficial for teams when performing complex tasks. In particular, general learning goals were most beneficial because they enabled teams to coordinate better, since members were less constrained to acquiring specific learning or performance outcomes in a rigid way.

Building on this work, we ask how disclosure of different types of ETGs affects team outcomes beyond just team performance. For example, when a team member discloses ETGs related to learning, are they perceived differently from those who disclose ETGs related to more instrumental outcomes, in terms of the extent to which others enjoy working with them or wish to continue working with them in the future? We speculated that when team members are attempting to gain a specific performance outcome from their team interactions, this instrumental behavior could be perceived as using their team members as a means to an end. If so, disclosure of more instrumental ETGs could harm team outcomes as a result of these negative interpersonal perceptions. To explore this possibility, we compare the disclosure of two main types of ETGs: those that are presented as general learning goals (Learning ETGs) and those that are presented as outcome goals in which the team is being used instrumentally (Instrumental ETGs). Prior work on relational aspects of instrumental goal pursuit (Orehek and Forest, 2016) has shown that individuals are motivated by viewing others as instrumental when that benefit is mutual, but can be damaging to the relationship if not. Recent qualitative findings also suggest that instrumental ETG disclosure can be risky, finding that team members feel “icky” when others disclose instrumental motives for joining a team (Sackett, 2017). Building on that work, we predicted that the disclosure of ETGs presented as learning goals would lead to more positive effects on team performance, team viability, and team satisfaction, compared with the disclosure of ETGs that are not learning goals.

Hypothesis 1: Disclosure of ETGs that involve a learning goal will elicit more positive effects on team performance, team viability, and team satisfaction than disclosure of ETGs without a learning goal component.

Research in multiple streams has investigated the presence of multiple goal types on performance outcomes for individuals and teams, with different conclusions. Research and theorizing about mixed motive situations suggests that disclosing ETGs that combine elements of learning as well as instrumentality might have less positive effects than ETGs that emphasize learning alone. For example, Feiler et al. (2012) found that making salient multiple motives for donation in a fundraising context led to lower levels of donation than did making salient any single motive. Research has also shown that introducing extrinsic rewards can undermine intrinsic motivation (Deci et al., 1999), another example of how mixing goal types can affect performance and motivational outcomes. On the other hand, research on creativity has shown that having multiple and potentially conflicting goals (for example, novelty and utility) can be beneficial for creative performance (Butler et al., 2003; Miron-Spektor and Erez, 2017). We predict that the findings of the mixed-motives research would be more relevant for the phenomenon of ETG awareness. Whereas the multiple goals in the case of this creativity research are directly related to the focal task, ETGs held by particular members of the team instead reflect information about the target, and so we would expect the effects of that to have more impact on interpersonal perceptions than eliciting cognitive stimulation. Therefore, we predicted an interaction effect such that ETGs that expressed both learning and instrumental goals (compared with just as learning goals) would lead to less positive effects on team performance, viability, and satisfaction.

Hypothesis 2: Disclosure of extra-team goals that mix goal types will elicit less positive effects on team performance, team viability, and team satisfaction than Learning-only ETGs.

### Perceptions of Commitment and Trust as Potential Mediating Mechanisms

We expect that the effects of ETG disclosure on team outcomes will be mediated through perceptions of the discloser’s team commitment and through trust of the discloser. There are reasons to predict that ETG disclosure could lead to either increased or decreased perceptions of commitment and trust. For example, a team member’s ETG to get promoted could lead others to perceive her as even more committed to the team and trust her more, because better outcomes for the team would help further her chances for promotion. On the other hand, disclosing such a goal could also lead team members to see her as less committed to the team and less trustworthy, since she plans to leave and is thus less invested in the long-term success of the team. Given that both trust and commitment are known antecedents of team performance and processes (Dirks, 1999; Levine and Moreland, 2002; Ilgen et al., 2004) we speculate that they would drive the effects of ETG disclosure regardless of whether ETG disclosure increases or decreases perceptions of commitment and trust – both in general and different types – on team performance, team viability, and team satisfaction.

Hypothesis 3: The effects of ETG disclosure on team performance, viability, and satisfaction are mediated by perceptions of the goal discloser’s commitment to the team’s goals, and trust in the goal discloser.

## Methods

### Empirical Context

The primary purpose of this laboratory experiment was to test the effects of the disclosure of ETGs by one team member on the team outcome and the experience of other team members. First, we tested whether the disclosure of an ETG by one team member (the target) positively or negatively affected important indicators of team effectiveness, such as team performance, team viability, and team satisfaction. We examined both the effects of ETG disclosure in general (regardless of the type of ETG content) as well as the effects of disclosing ETGs that vary in the extent to which they exhibit elements of learning goals and instrumental goals. We also explored the indirect effects of ETG disclosure on team performance, team viability, and team satisfaction by examining two potential mediators: perceptions of target commitment to the team goal and trust in the target.

To maximize ecological validity within an experimental paradigm, we sought to create a realistic and engaging team context for our student sample. From the office of Student Affairs at our university, we learned that first year undergraduate students face a number of challenges as they adjust to being a college student, particularly in a competitive environment such as the university where the study took place. These social and academic pressures are new for many of these students, and helping them to adjust to these new challenges was a major concern for the office of Student Affairs at the time of this study. Thus, the team task in our experiment was to brainstorm ideas for mobile apps that could be developed by the office of Student Affairs to help improve the first year student experience; the office of Student Affairs cooperated in this research, and evaluated the teams’ ideas (See Task and Procedure section for a detailed description of the study procedure). To enhance experimental control, we used confederate research assistants, posing as study participants, who were blind to study hypotheses, and trained to behave neutrally and similarly across conditions. At a designated point in the team interaction (prior to the start of the brainstorming task), the confederate made a statement that served as the experimental manipulation, which varied depending on which of the five conditions the team was assigned (see exact wording in the Task and Procedure section).

### Sample

Participants included 202 undergraduate and graduate students from a private university in the Southeastern United States (65% Female, 51% Undergraduate, *M*_*age*_ = 21.99, SD = 2.82), and were grouped into 65 teams (based on the time slot for which they signed up) across five conditions. Thirteen teams were in the control condition, 14 were in the Learning ETG condition, 13 were in the Instrumental ETG condition, 12 were in the mixed ETG condition, and 13 were in the Positive Statement condition, Each participant was paid $12 for participating in the study. Manipulation checks and debriefs revealed that 12 participants were suspicious of the confederate, so they were removed from analyses, leaving a final sample size of 190^1^.

### Task and Procedure

Teams of 3–5 members, which included a confederate team member played by an undergraduate research assistant, were assigned to one of five experimental conditions. In order to expedite data collection, two confederates were trained. Both were Caucasian, female, sophomore undergraduate students who were trained to present a very specific demeanor during these sessions: they were trained to participate in a generally pleasant, agreeable manner – making one or two positive statements throughout the team’s discussion, and making one or two contributions to the discussion, but never arguing with others, and never pushing too hard for an idea. Through extensive pilot testing, and debriefing sessions for participants in the control condition, we established that participants found the confederates’ behavior to be that of an “average” participant – not a leader, but also not a social loafer. Confederates were trained to ensure that the only thing that varied in their behavior in the other conditions was the specific ETG they disclosed. Each confederate participated in an equal number of sessions for each experimental condition, so that we could ensure that differences between conditions were not confounded with a particular confederate. For this reason, we rotated assignment to experimental condition rather than randomly assigning them in order to ensure an equal assignment to each confederate. The identity of the confederate did not moderate any of the relationships in our analyses and is therefore not discussed further.

Participants were told that they would be engaging in a brainstorming task with their team. First, they completed a short survey, which included demographic questions and several measures of individual differences^2^. Next, they watched a 3-min video in which the university’s Vice President of Student Affairs described the importance of addressing the challenges of first year students, and set up the “call” for coming up with ideas for mobile apps to help improve the first year student experience. Participants were then told that they would have 3 min to independently brainstorm ideas for mobile apps before having some time to work together to decide on one final idea to submit as a team. They were informed that the final ideas would be judged by Student Affairs staff members, and the winning team would be awarded a bonus prize of $20 per person (in actuality, one team from each experimental condition would be chosen as a winner, so that participants had an equal chance of winning across conditions).

Prior to beginning their independent brainstorming, participants were instructed to introduce themselves to one another. The experimenter running the study briefly left the room during these introductions, at which time the confederate disclosed an ETG (the experimental manipulation) after sharing her name, which varied according to the experimental condition as follows:

Not Learning/Not Instrumental (Control): [No extra statement was made]

Learning Only: “I was actually just invited to be on a Student Affairs task force that’s focused on these issues, so the more I can learn from this, the better. I really want to do well on this.

Learning and Instrumental: “I am planning to run for student government, which will be great for my resume, and the more I can learn from this, the better I can do. I really want to do well on this.

Instrumental Only: “I am actually trying to get a full time summer job in this lab, so I’m trying to do lots of studies to get exposure. I really want to do well on this.

Positive Statement: “I really want to do well on this.”^3^

Following the introductions, participants had 3 min to brainstorm independently. Then, they had 7 min to work together to discuss and decide on a final idea to submit as a team. Each team collaborated through a Google document, into which they each pasted their own individual ideas (so that everyone could see the contributions of each member) and into which they typed up their final team submission^4^. After the 7 min had passed, participants completed a second survey, in which they were asked about their experience of working with each other member of the team, and their experience working with the team as a whole (more detailed descriptions of measures to follow). The survey ended with a manipulation check, and participants were then debriefed together as a team, and paid $12 (per individual) for participating.

### Manipulation Check

At the end of the survey, participants in the control condition were asked whether they noticed anything unusual about “Member 1” (the confederate was always in Seat #1). Participants in the treatment conditions were asked whether they remembered what Member 1 said during their introductions, and how they reacted. No one in the control condition reported any suspicions, but 12 participants in the treatment conditions reported being suspicious that the disclosure of the goal made them question whether she was involved with the research program. Those 12 participants were subsequently removed from analyses. In addition to allowing us to screen suspicious participants, the manipulation checks also gave us confidence that participants in the treatment conditions had, indeed, noticed the disclosure of the ETG by the confederate; 80% of these participants were able to recall the ETGs that were disclosed^5^.

### Dependent Measures

#### Team Performance

The final ideas submitted by each team were judged independently by four Student Affairs executives (VP and Director level) who were blind to experimental condition as well as to the hypotheses being tested. They judged each team’s idea on four elements: feasibility, novelty, potential impact, and depth of development with 5 possible points for each element (a total of 20 points). These executives occupy high level positions at the university at which the study took place and work on issues related to student mental health, student life, and marketing, and therefore have the relevant expertise necessary to assess the feasibility, novelty, potential impact and depth of development of ideas for mobile apps to improve first year student experience. To assess inter-rater reliability, we used the r_*wg*_*_(j)_* index of within-group agreement (James et al., 1984) and there was strong support for within-group agreement in rater assessments of team performance; the median r_*wg*_*_(j)_* was equal to 0.87. Thus, there was sufficient agreement to average the independent ratings of the four expert raters, and we created a composite team performance measure combining all four elements.

All other team level measures were created by aggregating individual responses to self-report measures. These questions used Likert type scales with ratings from 1 (strongly disagree) to 7 (strongly agree). To justify aggregation to the team level, we examined two versions of the intraclass correlation coefficient (ICC): ICC(1) which demonstrates non-independence (i.e., that a significant portion of variance is attributable to team membership), ICC(2), which is similar to ICC(1) but accounts for team size, and the r*_*wg(j)*_* index of within-group agreement. These metrics are used to verify that there is a significant portion of variance explained by group membership – in other words, there is enough agreement within a group on a particular measure to claim that something unique is going on at the group level and aggregate responses to the group level (Bliese, 2000). ICC(1) and ICC(2) values were significant, indicating non-independence; median r*_*wg(j)*_* values were greater than or equal to 0.93, indicating high within-group agreement. Therefore, we used the team mean to operationalize our predictors at the team level. See Table 1 for a summary of aggregation indices.

**TABLE 1:** Descriptive statistics, aggregation indices, and team level correlations.

**Variable**	**M**	**SD**	**ICC(1)**	**ICC(2)**	**R*_*wg(j)*_***	**1**	**2**	**3**
1. Positive								
2. ETG	−	−	−	−	−	−		
3. Learning	−	−	−	−	−	−	−	−
4. Instrumental	−	−	−	−	−	−	−	−
5. Team Size	4.19	0.81	−	−	−	0.029	0.031	–0.194
6. Commitment (of Target)	6.03	0.56	0.07	0.18	0.95	–0.077	0.338**	0.289*
7. Trust (in Target)	5.33	0.84	0.23	0.46	0.93	0.013	0.077	0.087
8. Team Viability	5.51	0.80	0.13	0.3	0.93	0.019	0.254*	0.211*t*
9. Team Satisfaction	5.68	0.72	0.13	0.31	0.95	0.076	0.217*t*	0.207*t*
10. Team Performance	13.54	1.24	−	−	−	0.299*	0.023	0.132

**Variable**	**4**	**5**	**6**	**7**	**8**	**9**	**10**	

1. Positive								
2. ETG								
3. Learning								
4. Instrumental	−							
5. Team Size	0.252*	−						
6. Commitment (of Target)	0.157	–0.105	−					
7. Trust (in Target)	–0.086	–0.192	0.655***	−				
8. Team Viability	0.114	–0.086	0.609***	0.544***	−			
9. Team Satisfaction	0.113	–0.127	0.575***	0.537***	0.919***	−		
10. Team Performance	0.027	–0.046	–0.110	0.017	0.105	0.167	−	

*N* = 65 teams. t *p* < 0.10; * *p* < 0.05; ** *p* < 0.01; *** *p* < 0.001 (two-tailed).

#### Team Viability

Three items (slightly modified from Lewis, 2003) measured team viability (example item: “If it were up to me to continue working on this team, I would do it”). The three items were averaged to create a composite score (Cronbach’s alpha = 0.94).

#### Team Satisfaction

Three items (slightly modified from Gladstein, 1984) measured team satisfaction (example item: “I am very satisfied working in this team”). The three items were averaged to create a composite score (Cronbach’s alpha = 0.94).

### Mediators

#### Perceived Target Goal Commitment

Three items from Klein et al. (2001) goal commitment scale measured the extent to which participants perceived the target (confederate) to be committed to the team’s goal (example item: “XX is committed to pursuing the team’s goal”). The three items were averaged to create a composite measure (Cronbach’s alpha = 0.90).

#### Trust in Target

Three items modified from Mayer and Davis (1999) trust scale measured the extent to which participants trusted the target (confederate) (example item: “How willing are you to rely on XX”). The three items were averaged to create a composite measure (Cronbach’s alpha = 0.94).

### Control

Although team size was relatively homogeneous across our sample, with all teams comprising between 3 and 5 members (Mean = 4.3, SD = 0.76), we did find significant differences in team size among our four conditions (just due to the randomness of the signup process). Given this, we included team size (grand mean centered) as a covariate in all of our analyses. Results without team size as a covariate are nearly identical except that the effects are slightly stronger without the covariate in some cases. See Supplementary Appendix B for the results without the team size covariate.

### Additional Variables Collected in the Post-survey

This experiment was part of a doctoral dissertation and several additional measures were collected for robustness; we included measures of mood (positive and negative affect), willingness to work with the confederate in the future, perceptions that the confederate unfairly benefitted from participating in the task, and enjoyment working with the confederate. None of these measures produced any significant findings as dependent variables, nor did they produce consistent effects as moderators on any of our main dependent variables and thus are not discussed further. However, we report them here for the sake of transparency.

## Results

Descriptive statistics, agreement indices, and correlations among our team level variables are shown in Table 1.

### Analytic Approach

To assess the direct effects of ETG disclosure on team performance, team viability, and team satisfaction, we used OLS regression in R, controlling for team size (grand mean centered). The control condition is the reference group for all regressions. In order to partition out the effects of the positive statement contained in the three ETG disclosure conditions, we include a factor for “Positive Statement” in our models, which allows us to test the effects of ETG disclosure above and beyond the effects of the embedded positive statement^6^. To assess the indirect effects of ETG disclosure, we included perceptions of target’s commitment and trust in the target in the model as mediators. These predictors were grand mean centered, in order to improve the interpretability of the coefficients (Hofmann and Gavin, 1998). We then used the PROCESS macro for SPSS by Hayes (2012), which uses bootstrapped confidence intervals to analyze the indirect effects of ETG disclosure on team performance, team viability, and team satisfaction via perceptions of target’s commitment and trust in the target.

Our results are presented in two separate sections. First, we present results of analyses regarding the effects of disclosing ETGs in general (regardless of their content) on our three dependent variables (team performance, team viability, and team satisfaction), which speak to Research Question 1. Then, we present results of analyses regarding the effects of different types of ETG disclosure (Learning ETGs, Instrumental ETGs, and combined) on the same three dependent variables to test Hypotheses 1 and 2. In each section, we also include exploratory mediation analyses in which we investigate whether perceptions of target’s commitment to the team’s goal and trust in the target mediate these effects, to test Hypothesis 3.

### The Direct and Indirect Effects of ETG Disclosure

#### Team Performance

Table 2 summarizes the results of the team performance regression models. In Model 1, we regressed Positive Statement on team performance, controlling for team size (grand mean centered), which jointly predict team performance, *R^2^* = 0.062. The findings indicate that there was a significant, positive effect of making a positive statement (B = 0.94, SE = 0.3, *p* = 0.017) on team performance. In Model 2 we added in ETG disclosure in general, which resulted in a significant change in *R^2^*,Δ*R*^2^ = 0.05, *p* = 0.035. Again we found a significant, positive effect of making a positive statement (B = 1.54, SE = 0.47, *p* = 0.002) and found a significant, positive, effect of ETG disclosure (B = 0.81, SE = 0.37, *p* = 0.034) on team performance above and beyond the effect of making a positive statement. In Model 3, we added in the two potential mediators of commitment and trust, which resulted in a small but non-significant change in *R^2^*,Δ*R*^2^ = 0.03, *p* = 0.11. In this model, Positive statement and ETG disclosure retained their positive effects, and there was also a significant, negative effect of commitment (B = −0.78, SE = 0.37, *p* = 0.04), but no significant effect of trust. We then performed a test of mediation for both potential mediators simultaneously, with ETG disclosure as the predictor (while controlling for team size as well as the effect of making a positive statement), using the PROCESS macro for SPSS (Hayes, 2012: model 4). Perceived commitment partially, and negatively, mediated the effects of ETG disclosure on Team Performance, indirect effect = −0.42, SE = 0.24, 95% CI = [−1.04, −0.05]. However, there was no significant mediation for trust, indirect effect = 0.08, SE = 0.11, 95% CI = [−0.06, 0.44].

**TABLE 2:** Regressions – ETG disclosure on team performance.

	***Dependent variable:* Team Performance**					
	**(1)**	**(2)**	**(3)**	**(4)**	**(5)**	**(6)**
Team Size	–0.069	–0.093	–0.107	–0.027	–0.031	–0.027
	(0.187)	(0.182)	(0.183)	(0.193)	(0.195)	(0.193)
Positive Statement	0.939*	1.544**	1.689**	1.460**	1.539**	1.549**
	(0.384)	(0.466)	(0.463)	(0.436)	(0.468)	(0.429)
ETG		0.807*	1.154**			
		(0.373)	(0.403)			
Commitment			−0.780*			−0.782*
			(0.373)			(0.369)
Trust			0.293			0.339
			(0.234)			(0.237)
Learning ETG				0.751*	0.900*t*	0.963**
				(0.329)	(0.453)	(0.338)
Instrumental ETG				0.272	0.437	0.462
				(0.337)	(0.481)	(0.342)
Learning* Instrumental					–0.313	
					(0.649)	
Constant	13.651**	13.149**	12.959**	12.959**	12.897**	12.771**
	(0.797)	(0.809)	(0.811)	(0.843)	(0.858)	(0.842)
Adjusted R^2^	0.062*t*	0.115*	0.149*	0.121*	0.109*	0.157*
Model Comparison	−	1–2	2–3	1–4	4–5	4–6
ΔR^2^	−	0.05*	0.03	0.06*t*	–0.01	0.04

*N* = 64; t *p* < 0.1; *p < 0.05; **p < 0.01; continuous predictor variables grand mean centered.

#### Team Viability

Table 3 summarizes the results of the team viability regression models. In Model 1, we started by regressing the positive statement factor on team viability, controlling for team size (grand mean centered). The model did not predict team viability, *R*^2^ = −0.02, and we found no significant effect (B = 0.04, SE = 0.25, *p* = 0.869). In Model 2 we added in ETG disclosure, which resulted in a significant change in *R^2^*,Δ*R*^2^ = 0.11, *p* = 0.005. Here we find a marginally significant effect for the positive statement factor (B = 0.57, SE = 0.31, *p* = 0.06), and a significant, positive effect of ETG disclosure (B = 0.71, SE = 0.25, *p* = 0.006) on team viability. In Model 3, we added in the two potential mediators of commitment and trust, which resulted in a large, significant change in *R^2^*,Δ*R*^2^ = 0.31, *p* = 0.000. In this model, Positive statement and ETG disclosure no longer retained their positive effects, and there were significant, positive effects of both commitment (B = 0.51, SE = 0.20, *p* = 0.014), and trust (B = 0.28, SE = 0.13, *p* = 0.035). We then performed a test of mediation for both potential mediators simultaneously, with ETG disclosure as the predictor (while controlling for team size as well as the effect of making a positive statement), using the PROCESS macro for SPSS (Hayes, 2012: model 4). Results showed that perceived commitment mediated the effects of ETG disclosure on Team Viability, indirect effect = 0.28, SE = 0.16, 95% CI = [0.05, 0.72]. However, there was no significant mediation for trust, indirect effect = 0.07, SE = 0.09, 95% CI = [−0.06, 0.32].

**TABLE 3:** Regressions – ETG disclosure on team viability.

	***Dependent variable:* Team Viability**					
	**(1)**	**(2)**	**(3)**	**(4)**	**(5)**	**(6)**
Team Size	–0.086	–0.107	–0.005	–0.086	–0.096	–0.006
	(0.126)	(0.119)	(0.099)	(0.131)	(0.129)	(0.107)
Positive Statement	0.042	0.572*t*	0.352	0.383	0.570*t*	0.353
	(0.252)	(0.302)	(0.252)	(0.292)	(0.307)	(0.256)
ETG		0.705**	0.356			
		(0.247)	(0.222)			
Commitment			0.514*			0.509*
			(0.204)			(0.209)
Trust			0.277*			0.283*
			(0.129)			(0.133)
Learning ETG				0.400*t*	0.755*	0.335
				(0.225)	(0.303)	(0.265)
Instrumental ETG				0.278	0.671*	0.348
				(0.231)	(0.322)	(0.276)
Learning* Instrumental					−0.747*t*	–0.292
					(0.435)	(0.365)
Constant	5.864**	5.423**	5.248**	5.521**	5.375**	5.253**
	(0.537)	(0.531)	(0.443)	(0.572)	(0.570)	(0.474)
Adjusted R^2^	–0.024	0.082*	0.387**	0.021	0.052	0.366**
Model Comparison	−	1–2	2–3	1–4	4–5	5–6
ΔR^2^	−	0.11*	0.31***	0.05*	0.03*t*	0.31***

*N* = 65; t *p* < 0.1; *p < 0.05; **p < 0.01; ***p < 0.001; continuous predictor variables grand mean centered.

#### Team Satisfaction

Table 4 summarizes the results of the team satisfaction regression models. In Model 1, we started by regressing the positive statement factor on team satisfaction. The model did not predict team satisfaction, *R^2^* = −0.009, and we found no significant effect (B = 0.14, SE = 0.22, *p* = 0.530). In Model 2 we added in General ETG disclosure, which resulted in a significant change in *R^2^*,Δ*R*^2^ = 0.11, *p* = 0.006. Here we find a significant effect for the positive statement factor (B = 0.62, SE = 0.27, *p* = 0.025), and a significant, positive effect of ETG disclosure (B = 0.63, SE = 0.22, *p* = 0.006) on team satisfaction. In Model 3, we added in the two potential mediators of commitment and trust, which resulted in a large, significant change in *R^2^*,Δ*R*^2^ = 0.27, *p* = 0.000. In this model, Positive statement and ETG disclosure retained marginally significant positive effects, and there were significant, positive effects of both commitment (B = 0.40, SE = 0.19, *p* = 0.037), and trust (B = 0.26, SE = 0.12, *p* = 0.029). We then performed a test of mediation for both potential mediators simultaneously, with ETG disclosure as the predictor (while controlling for team size as well as the effect of making a positive statement), using the PROCESS macro for SPSS (Hayes, 2012: model 4). Results showed that perceived commitment mediated the effects of ETG disclosure on Team Satisfaction, indirect effect = 0.21, SE = 0.14, 95% CI = [0.001, 0.56]. However, there was no significant mediation for trust, indirect effect = 0.07, SE = 0.08, 95% CI = [−0.05, 0.28].

**TABLE 4:** Regressions – ETG disclosure on team satisfaction.

	***Dependent variable:* Team Satisfaction**					
	**(1)**	**(2)**	**(3)**	**(4)**	**(5)**	**(6)**
Team Size	–0.115	–0.134	–0.045	–0.122	–0.129	–0.047
	(0.112)	(0.106)	(0.091)	(0.116)	(0.115)	(0.096)
Positive Statement	0.142	0.617*	0.436*t*	0.487*t*	0.616*	0.404*t*
	(0.224)	(0.269)	(0.229)	(0.257)	(0.273)	(0.213)
ETG		0.632**	0.351*t*			
		(0.220)	(0.202)			
Commitment			0.396*			0.389*
			(0.186)			(0.185)
Trust			0.262*			0.282*
			(0.117)			(0.119)
Learning ETG				0.377*t*	0.621*	0.213
				(0.199)	(0.270)	(0.171)
Instrumental ETG				0.311	0.581*	0.252
				(0.203)	(0.286)	(0.173)
Learning*Instrumental					–0.513	
					(0.387)	
Constant	6.133**	5.738**	5.571**	5.819**	5.718**	5.612**
	(0.478)	(0.473)	(0.404)	(0.504)	(0.507)	(0.421)
Adjusted R^2^	–0.009	0.097*	0.368**	0.057	0.069	0.364**
Model Comparison	−	1–2	2–3	1–4	4–5	4–6
ΔR^2^	−	0.11**	0.27***	0.07*	0.01	0.30***

**N* = 65; t *p* < 0.1; ^∗^*p* < 0.05; ^∗∗^*p* < 0.01; ^∗∗∗^*p* < 0.001; continuous predictor variables grand mean centered.*

Results explained above suggest that ETG disclosure, in general, has positive effects on team performance, team viability, and team satisfaction, and that those effects are partially or fully mediated by perceptions of the target’s commitment to the team’s goal (negatively, in the case of team performance). In the next section, we explore whether disclosing different types of ETGs (Learning ETGs, Instrumental ETGs, and those that combine elements of both) have differential effects on our three outcomes of interest, to test Hypotheses 1, 2, and 3.

### The Direct and Indirect Effects of Disclosing Different Types of ETGs

#### Team Performance

**FIGURE 1:**
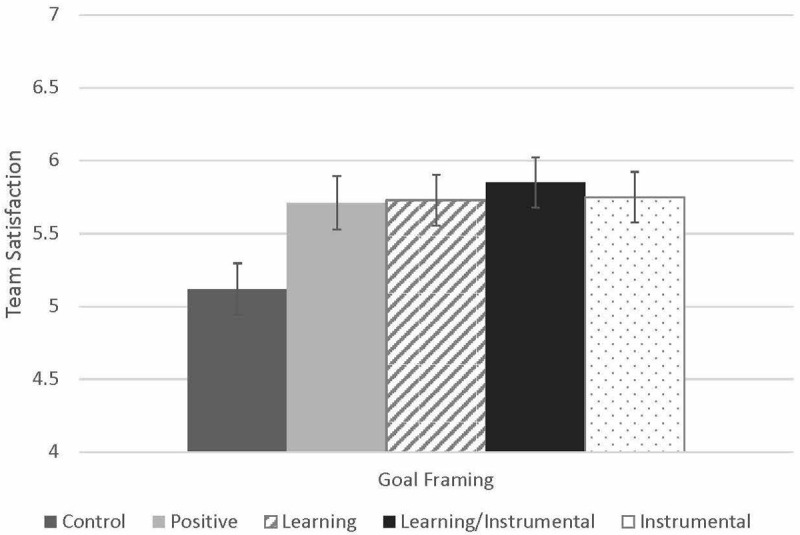
Effects of goal type on team satisfaction. Controls for team size.

Table 2 shows a summary of the results of the team performance regression models. Figure 1 shows the pattern of group means across the five conditions for team performance conditions (note that these figures are intended to provide a visual overview of means by condition; in the analyses to follow, we examine the effects of learning, instrumental, and mixed factors, rather than cell comparisons by condition). We test Hypothesis 1 for team performance in Model 4. To do this, we built on Model 1 (described earlier) and added Learning ETG disclosure and Instrumental ETG disclosure, resulting in a marginally significant change in *R^2^*,Δ*R*^2^ = 0.06, *p* = 0.054. We again found a significant positive effect of making a positive statement (B = 1.46, SE = 0.44, *p* = 0.001), as well as a positive effect of Learning ETG disclosure, over and above the effect of making a positive statement (B = 0.75, SE = 0.33, *p* = 0.026), but no significant effect of Instrumental ETG disclosure (B = 0.27, SE = 0.34, *p* = 0.422). Next, we tested Hypothesis 2 for team performance by adding an interaction term for Learning ETG disclosure and Instrumental ETG disclosure in Model 5, which did not result in a significant change in *R^2^*,Δ*R*^2^ = −0.01, *p* = 0.631, and we found no significant interaction effect. In Model 6 we added commitment and trust to the model without the interaction term, which resulted in a small but non-significant change in *R^2^*,Δ*R*^2^ = 0.04, *p* = 0.11. We found a significant, negative effect of commitment (B = −0.78, SE = 0.37, *p* = 0.038) but no significant effects for trust (B = 0.34, SE = 0.24, *p* = 0.158). To test Hypothesis 3 for team performance we performed a test of mediation for both potential mediators simultaneously, with Learning ETG disclosure as the predictor (while controlling for team size as well as the effect of making a positive statement), using the PROCESS macro for SPSS (Hayes, 2012: Model 4). Here, perceived commitment partially, and negatively, mediated the effects of Learning ETG disclosure on Team Performance, indirect effect = −0.22, SE = 0.17, 95% CI = [−0.65, −0.002]. However, there was no significant mediation for trust, indirect effect = 0.03, SE = 0.09, 95% CI = [−0.09, 0.30].

#### Team Viability

**FIGURE 2:**
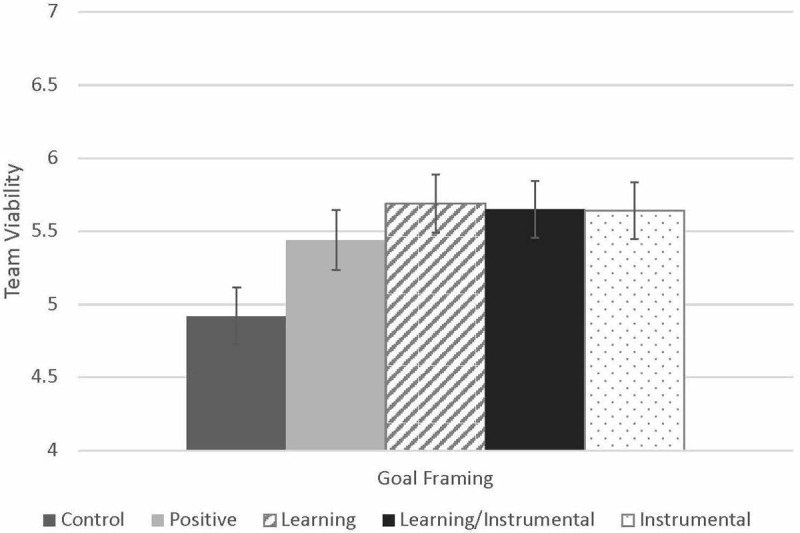
Effects of goal type on team viability. Controls for team size.

Table 3 presents a summary of the results of the team viability regression models. Figure 2 shows the pattern of group means across the five conditions for team viability. We test Hypothesis 1 for team viability in Model 4. To do this, we built on Model 1 (described earlier) and added Learning ETG disclosure and Instrumental ETG disclosure, resulting in a marginally significant change in *R^2^*,Δ*R*^2^ = 0.05, *p* = 0.096. Here we found no significant effect of making a positive statement (B = 38, SE = 0.29, *p* = 0.194), a marginally significant, positive effect of Learning ETG disclosure (B = 0.40, SE = 0.23, *p* = 0.081), and no significant effect of Instrumental ETG disclosure (B = 0.28, SE = 0.23, *p* = 0.232). Next, we tested Hypothesis 2 for team viability in Model 5, by adding an interaction term for Learning ETG disclosure and Instrumental ETG disclosure, which resulted in a small, marginally significant change in *R^2^*,Δ*R*^2^ = 0.03, *p* = 0.091. In this model, we found a marginally significant effect of the positive statement factor (B = 0.57, SE = 0.31, *p* = 0.068), a significant main effect of Learning ETG disclosure (B = 0.76, SE = 0.30, *p* = 0.016), and a significant main effect of Instrumental ETG disclosure (B = 0.67, SE = 0.32, *p* = 0.042). Analyses also revealed a marginally significant interaction effect (B = −0.75, SE = 0.44, *p* = 0.091), suggesting that ETGs that contain elements of both learning and instrumentality lead to marginally lower perceptions of team viability. In Model 6 we added commitment and trust to the model with the interaction term, which resulted in a large and significant change in *R^2^*,Δ*R*^2^ = 0.31, *p* = 0.000. We found a significant, positive effect of commitment (B = 0.51, SE = 0.21, *p* = 0.018) and trust (B = 0.28, SE = 0.13, *p* = 0.038). To test Hypothesis 3 for team viability we performed a test of moderated mediation with both potential mediators simultaneously, to test the degree to which perceptions of commitment and trust mediate the relationship between Learning ETG disclosure and team viability with and without elements of Instrumental ETG disclosure (while controlling for team size as well as the effect of making a positive statement), using the PROCESS macro for SPSS (Hayes, 2012: Model 7). This analysis revealed that perceived commitment mediated the effects of Learning ETG disclosure on team viability, but only when those Learning ETGs did not include elements of instrumentality (conditional indirect effect = 0.26, SE = 0.15, 95% CI = [0.04, 0.63]). When Learning ETGs also contained elements of instrumentality, perceived commitment did not mediate the effects of disclosure on team viability (conditional indirect effect = 0.03, SE = 0.15, 95% CI = [−0.27, 0.38]). There was no significant mediation for trust, neither for Learning ETGs without instrumentality (conditional indirect effect = 0.08, SE = 0.08, 95% CI = [−0.02, 0.30]) or with instrumentality (conditional indirect effect = −0.058, SE = 0.11, 95% CI = [−0.33, 0.11]).

#### Team Satisfaction

**FIGURE 3:**
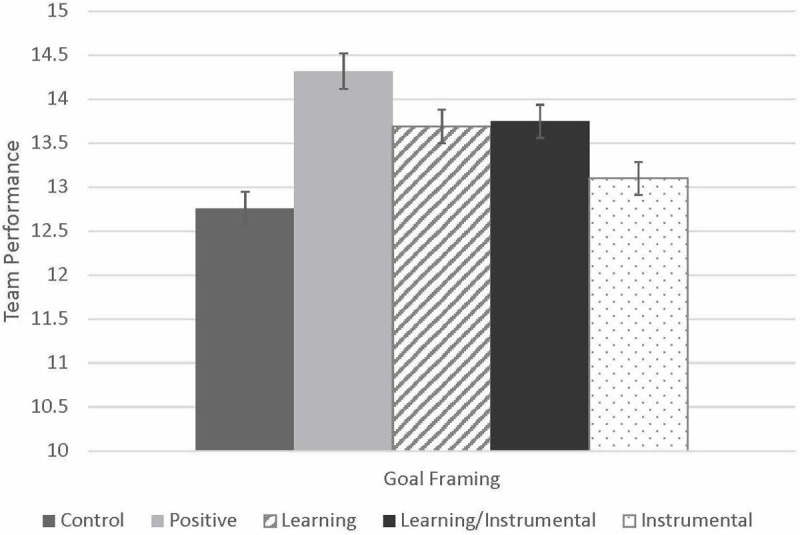
Effects of goal type on team performance. Controls for team size.

Table 4 presents a summary of the results of the team satisfaction regression models. Figure 3 shows the pattern of group means across the five conditions for team viability. In Model 4, we test Hypothesis 1 for team satisfaction. To do this, we built on Model 1 (described earlier) and added Learning ETG disclosure and Instrumental ETG disclosure, resulting in a significant change in *R^2^*,Δ*R*^2^ = 0.07, *p* = 0.049. We found a marginally significant positive effect of making a positive statement (B = 0.49, SE = 0.26, *p* = 0.063), as well as a marginally significant, positive effect of Learning ETG disclosure, over and above the effect of making a positive statement (B = 0.38, SE = 0.20, *p* = 0.063), but no significant effect of Instrumental ETG disclosure (B = 0.31, SE = 0.20, *p* = 0.131). Next, we tested Hypothesis 2 for team satisfaction by adding an interaction term for Learning ETG disclosure and Instrumental ETG disclosure in Model 5, which did not result in a significant change in *R^2^*,Δ*R*^2^ = 0.01, *p* = 0.189. In this model, we found a significant effect of the positive statement factor (B = 0.62, SE = 0.27, *p* = 0.028), a significant main effect of Learning ETG disclosure (B = 0.62, SE = 0.27, *p* = 0.025), and a significant main effect of Instrumental ETG disclosure (B = 0.58, SE = 0.29, *p* = 0.047), but no interaction effect (B = −0.51, SE = 0.39, *p* = 0.189). In Model 6 we added commitment and trust to the model without the interaction term, which resulted in a large and significant change in *R^2^*,Δ*R*^2^ = 0.30, *p* = 0.000. Here Learning ETG disclosure and Instrumental ETG disclosure no longer retained their positive effects and we found a significant, positive effect of commitment (B = 0.39, SE = 0.19, *p* = 0.040) and trust (B = 0.28, SE = 0.21, *p* = 0.021). To test Hypothesis 3 for team satisfaction, we performed a test of mediation with both potential mediators simultaneously, with Learning ETG disclosure as the predictor (while controlling for team size as well as the effect of making a positive statement), using the PROCESS macro for SPSS (Hayes, 2012: Model 4). Here, perceived commitment mediated the effects of Learning ETG disclosure on Team Satisfaction, indirect effect = 0.16, SE = 0.12, 95% CI = [0.006, 0.48]. However, there was no significant mediation for trust, indirect effect = 0.03, SE = 0.07, 95% CI = [−0.08, 0.21]. Finally, we performed a test of mediation with both potential mediators simultaneously, with Instrumental ETG disclosure as the predictor (while controlling for team size as well as the effect of making a positive statement), using the PROCESS macro for SPSS (Hayes, 2012: Model 4). Here, there was no mediation via perceived commitment, indirect effect = 10, SE = 0.10, 95% CI = [−0.02, 0.37] or via trust, indirect effect = −0.02, SE = 0.07, 95% CI = [−0.19, 0.09].

## Discussion

This study provides evidence that ETG disclosure, in general, may have positive effects on team performance, team viability, and team satisfaction, and that those effects are partially or fully mediated by perceptions of the target’s commitment to the team’s goal (negatively, in the case of team performance). The study also suggests that different types of ETG content differentially affect these outcomes. In particular, disclosing Learning ETGs, rather than Instrumental ETGs or both goals, may be particularly beneficial, in this academic context. In line with Hypothesis 1, the study found that Learning ETG disclosure had positive effects on team performance, team viability, and team satisfaction while Instrumental ETG disclosure did not have similar effects. The findings provided weak support for Hypothesis 2, revealing only a marginally significant interaction effect of Learning and Instrumental ETG disclosure on one of the three outcomes (team viability).

Thus, it seems that in general, it was beneficial for the target team member to disclose extra-team goals, but more beneficial to disclose learning, rather than instrumental, extra-team goals. In an effort to explore two possible mechanisms of these effects, we considered trust and commitment as potential mediators. Results for Hypothesis 3 were inconsistent. Trust in the target played no mediating role in any of the effects. In contrast, perceptions of the target’s commitment to the team’s goals had an indirect negative effect in the pathway from Learning ETG disclosure to team performance, but a positive effect for team satisfaction. For team viability, a moderated mediation also emerged, such that perceived commitment positively mediated the effects of Learning ETG disclosure, but only when those Learning ETGs did not include elements of instrumentality.

Given the mixed and inconsistent nature of the mediation results, we hesitate to over-weight the magnitude or generalizability of any of the findings. That being said, readers may be curious about the negative pathway played by perceptions of commitment in effects on performance. The mediation analyses suggest that, in addition to the positive effects of disclosing ETGs on team performance, there may also be detrimental downstream consequences of disclosing ETGs on team performance, mediated by increased perceptions of commitment to the team’s goal by the goal holder. This finding that increased perceptions of a goal holder’s commitment to the team’s goal may reduce team performance when ETGs are disclosed was surprising. It is possible that perceiving another team member as being highly committed to the team’s goal could result in reduced effort of the perceivers, as in social loafing (Latané et al., 1979; Karau and Williams, 1993). Qualitative comments from participants during the debrief do support the idea that participants saw the confederate as especially motivated in the Learning ETG condition, with comments such as “I thought she’d come up with good ideas,” and “good thing she is here to help.” However, no one made comments that suggested they intended to put in less effort as a result, so it is difficult to conclude definitively that such beliefs resulted in social loafing. It is also possible that this effect is specious, given that we tested the role of two mediators in three separate mediation analyses, and thus, the possibility for type one error is high. Finally, it is also possible that something about this specific ETG disclosure or team performance elicited this effect. Indeed, given the early stage of this research, generalizations of all findings here beyond the paradigm used are unwise at this point. Future research should examine effects of ETG disclosure on team processes and performance both to confirm and explain the positive overall effects of ETG disclosure, as well as to better understand the mechanisms behind any such effects.

Also surprising was that the study did not find any mediation of our ETG disclosure effects via trust in the goal holder for any of our dependent variables. We speculate that the lack of role for trust may stem from the short-term nature of the team interaction, as well as the type of task. More specifically, these were teams that were not going to be working together beyond the lab experiment, so there may have been no reason for trust to be meaningfully developed. In addition, participants were engaging in a creative task for which trust was not as relevant as it would be in other types of interdependence work that has higher stakes or a more objective solution where a specific performance outcome is required of each member.

### Limitations

In general, these experimental findings are consistent with recent qualitative work that highlights both the positive and negative effects of ETG disclosure (Sackett, 2017). However, this research suffers from several main limitations that we hope to address in future research. In particular, although we were able to disentangle the effects of ETG disclosure from any embedded positive statement, other potential alternatives and confounds may exist. For example, it is possible that disclosure of individuating information of any kind was responsible for the overall effects (although not the Learning vs. Instrumental effects). Future studies could compare the effects of individuating information that is not goal related (e.g., disclosure of skills or experience) with ETG disclosure. In addition, as mentioned earlier, the short duration of the team’s interactions with one another may also have limited the ability of constructs like trust to meaningfully emerge, and we do not know whether our findings are generalizable to other types of tasks or contexts. Future research can address these issues by examining these processes in the field, where teams are working together long term and on a variety of types of tasks. Finally, as we acknowledged in our theorizing, there are multiple paths to goal awareness, and disclosure is just one of them. We chose to operationalize awareness as disclosure partly because it can be cleanly manipulated in an experimental context. In our manipulation check, the disclosure did indeed result in participants being able to recall the ETGs disclosed (and therefore we are confident that awareness was achieved). However, future research that investigates how awareness that is the result of other pathways beyond disclosure would be fruitful. For example, it would be useful to know whether awareness that is based on inquiry, observation, second-hand information results in similar effects, Additional future directions are discussed below.

### Future Directions

By exploring the consequences of developing awareness about extra-team content – specifically goals – we build on the existing team cognition literature, which has previously considered a narrower set of “team-relevant” content such as members’ shared awareness of task interdependencies, roles, and team goals (Mathieu et al., 2000; DeChurch and Mesmer-Magnus, 2010) or expertise (Lewis, 2003). As team boundaries become increasingly fluid, and organizational work becomes more complex (Wageman et al., 2012), it is critical to understand the extent to which team members should expand their shared mental models to include information that was not previously considered relevant to the team’s interdependent work. Overall, our findings suggest that there may be benefits of disclosing ETGs to one’s team members, and that presenting one’s ETGs in a way that conveys them as learning goals, rather than as instrumental goals, may be a useful strategy, at least in the university environment. While this experiment makes a first contribution to exploring the effects of ETG disclosure on individual and team outcomes, it only begins to delve into the interpersonal dynamics that may unfold after ETG disclosure. Below we describe several possible avenues for future research.

#### Configurations of ETG Disclosure and Awareness at the Team Level

In this study, we examined how the disclosure of an ETG by a single team member affected the perceptions of the other team members, as well as team performance. However, there are also several avenues for future research to explore that involve varying the patterns of ETG disclosure as well as ETG awareness within a team. One possibility is to examine how different patterns of ETG disclosure may affect outcomes; for example, exploring what happens when different configurations of team members disclose ETGs (i.e., one member, two members, all members, etc.). This network approach would follow on recent calls for teams research to examine team cognition with a more compilational approach (Humphrey and Aime, 2014). Another direction is to explore what happens when different combinations of ETG types are disclosed within a team. Research has found that when team members have a mixture of different values regarding their approach to task interdependence, their performance can suffer (Wageman and Gordon, 2005). Future research could explore how performance is affected when some members disclose learning ETG while others disclose instrumental ETGs. A third direction is to examine how different configurations of awareness affect individual and team outcomes. Recent research has explored the effects of centralization of metacognitions related to transactive memory, and found teams with centralized metacognition to have a performance advantage (Mell et al., 2014). In this study, all members had awareness of the confederate’s ETG, but future research could explore whether centralized or decentralized ETG awareness differentially affects individual and team outcomes.

#### Misaligned ETGs

In this study, we focus exclusively on ETGs that are aligned with team goals in ways that are complementary. This is because the purpose of this study was to examine a situation in which goals are objectively aligned, but in which awareness of that alignment can be perceived negatively by other team members; such contexts are consistent with the type of goal alignment that was present in Sackett’s qualitative field study. However, it is just as likely that team members will have ETGs that conflict with the goals of the team, perhaps especially if their team membership is not voluntary. In the future, we could also explore situations in which goals are explicitly in conflict and test the effects of learning vs. instrumental ETG disclosure.

## Conclusion

This study contributes to research on interpersonal perception, team cognition, team goal pursuit, and team processes, to extend the focus of team cognition to content that extends beyond a team’s boundaries, and to better understand the processes through which team members may be able to benefit through the exchange of information about their broader goal systems. Given that people pursue many interconnected goals in their daily professional lives, this study provides evidence that awareness of these inter-goal relationships can indeed have consequences for team level outcomes, and warrant more attention moving forward.

## Data Availability Statement

The raw data supporting the conclusions of this article will be made available by the authors, without undue reservation.

## Ethics Statement

The studies involving human participants were reviewed and approved by Duke University Institutional Review Board. The patients/participants provided their written informed consent to participate in this study.

## Author Contributions

ES and GF contributed to conception and design of the study. ES collected the data, performed statistical analysis, and wrote the first draft of the manuscript. Both authors contributed to manuscript revision, read, and approved the submitted version.

## Conflict of Interest

The authors declare that the research was conducted in the absence of any commercial or financial relationships that could be construed as a potential conflict of interest.

## Footnotes

1 The main patterns and significance tests are similar if these suspicious participants were included in analyses. Results of the analyses including the suspicious participants are from the authors upon request.

2 The variables collected in this pre-survey included gender, student level (graduate vs. undergraduate), baseline commitment to addressing first year student issues, and measures of BIS/BAS. None of these variables produced consistent, significant findings with regard to moderation, and are therefore not discussed.

3 The fifth condition was run after completion of the other four conditions; it was added to help address a potential confound introduced by the use of “I really want to do well on this” in all treatment conditions. This condition was run in the same lab setting, in the same academic term, and with the same confederates, as the other conditions. For conceptual clarity, we include results from this condition in our analysis with the other four conditions here. Results from analyses of just the original four conditions are presented in Supplementary Appendix A.

4 To control for the quality of the confederate’s contributions, the confederate’s contribution needed to be the same each time. To generate ideas for the confederate, we conducted seven pilot team sessions (which included 19 participants). Participants in the pilot sessions produced, on average, four ideas in 3 min. Therefore, we had the confederate submit four ideas, and we selected four ideas that had been generated at least twice across participants in the pilot sessions, so that the confederate’s ideas would be relatively typical of ideas that participants would generate.

5 Even though 20% of participants (22 individuals) in the ETG treatment conditions did not explicitly recall the ETGs that were disclosed by the confederate in the manipulation check, all but 4 of them recalled other information provided by the confederate during her introduction, increasing confidence that they were paying attention. Explicit recall is a very high threshold for attention; recognition measures would have provided better tests and should be incorporated in any future research using this paradigm. Furthermore, research has shown that even when participants do not recall the manipulation, they can nonetheless be affected by it (Chartrand and Bargh, 1996). We found the pattern of results to be similar when we analyzed the data excluding the participants who did not explicitly recall the ETG disclosure.

6 We also conducted analyses without the positive statement condition to explore the effects of disclosing different ETGs compared with no statement (excluding the positive statement condition). The results do not change significantly when the positive statement condition is not included in the analyses. These results appear in the Supplementary Appendix.
