# Supplementary material for: The Effects of Extra-Team Goal Disclosure on Team Performance, Viability, and Satisfaction
Source: Front Psychol. 2021 Jan 12;11:548842. doi: 10.3389/fpsyg.2020.548842 (PMC7835427; doi:10.3389/fpsyg.2020.548842)
Supplement: Supplementary file 1 [file Table_1.DOCX]

**Supplementary Appendix A: Regression Tables and Mediation Analyses excluding the positive statement condition (analyses using the four original conditions only)**

As described in the methods section of this paper, this experiment initially had only four conditions. After running the first four conditions, it came to our attention that the statement “I really want to do well on this” that was appended to each of the treatment conditions created a potential confound. Specifically, this phrase could be interpreted as a positive signal of effort, which could potentially drive any effects of ETG disclosure in general and make it difficult to disentangle the effects of goal content from the effects of the positive statement. For this reason, we decided to run a fifth condition that included simply an assertion of effort, but no goal content, to help us distinguish between the effects of goal content and positive signaling of effort. This condition was run in the same lab setting, in the same academic term, and with the same confederates, as the other conditions, and thus seems comparable. For conceptual clarity, we presented the results of our original data collection and our supplemental condition together in the main body of this paper, which enables us to disentangle the effects of the positive statement from the goal content in our analyses. The results from analyses of just the original four conditions are presented in this supplementary appendix. Supplementary tables, including correlations (Table S1) and regression results (Tables S2-S4), appear following the results of our analyses.

**The Direct and Indirect Effects of General ETG disclosure**

To assess the direct effects of General ETG disclosure on team performance, team viability, and team satisfaction, we used OLS regression in R, controlling for team size (grand mean centered). To assess the indirect effects of General ETG disclosure, we included perceptions of target’s commitment and trust in the target in the model as mediators. These predictors were grand mean centered, in order to improve the interpretability of the coefficients (Hofmann & Gavin, 1998). We then used the PROCESS macro for SPSS by Hayes (Model 4, 2012), which uses bootstrapped confidence intervals to analyze the indirect effects of General ETG disclosure on team performance, team viability, and team satisfaction via perceptions of target’s commitment and trust in the target.

***Team performance.*** Table S2 summarizes the results of the team performance regression models. We ran a series of regressions with the four original conditions, using the original control condition as the reference group, controlling for team size (grand mean centered). In Model 1, we regressed ETG disclosure in general on team performance, controlling for team size (grand mean centered), which jointly predict team performance, *R^2^* = .061. There was a significant, positive effect of general ETG disclosure (B = .81, SE = .36, *p* =.030) on team performance. In Model 2, we added in the two potential mediators of commitment and trust, which resulted in a small but nonsignificant change in *R^2^,* Δ*R^2^* = .05, *p* = .118. In this model, ETG disclosure retained its positive effects, and there was also a significant, negative effect of commitment (B = -.79, SE = .37, *p* =.040), but no significant effect of trust (B = .30, SE = .24, *p* = .211). We then performed a test of mediation for both potential mediators simultaneously, with general ETG disclosure as the predictor (while controlling for team size), using the PROCESS macro for SPSS (Hayes, 2012: model 4). Perceived commitment partially, and negatively, mediated the effects of ETG disclosure on Team Performance, indirect effect = -.43, SE = .26, 95% CI = [-1.07, -.05]. However, there was no significant mediation for trust, indirect effect = .08, SE = .12, 95% CI = [-.06, .44].

***Team Viability.*** Table S3 summarizes the results of the team viability regression models. We ran a series of regressions with the four original conditions, using the original control condition as the reference group, controlling for team size (grand mean centered). In Model 1, we regressed general ETG disclosure on team viability, controlling for team size (grand mean centered), which predicted a significant amount of variance, *R^2^* = .15, *p* = .007. We found a significant positive effect of General ETG disclosure (B = .71, SE = .23, *p* = .003) on team viability. In Model 2, we added in the two potential mediators of commitment and trust, which resulted in a large, significant change in *R^2^,* Δ*R^2^* = .24, *p* <=.000. In this model, ETG disclosure no longer retained its significant effects, and there were significant or marginally significant, positive effects of both commitment (B = .42, SE = .20, *p* = .045), and trust (B = .24, SE = .13, *p* = .067). We then performed a test of mediation for both potential mediators simultaneously, with general ETG disclosure as the predictor (while controlling for team size), using the PROCESS macro for SPSS (Hayes, 2012: model 4). Results showed that perceived commitment mediated the effects of ETG disclosure on Team Viability, indirect effect = .23, SE = .15, 95% CI = [.002, .63]. However, there was no significant mediation for trust, indirect effect = .06, SE = .08, 95% CI = [-.06, .28].

***Team Satisfaction.*** Table S4 summarizes the results of the team satisfaction regression models. We ran a series of regressions with the four original conditions, using the original control condition as the reference group, controlling for team size (grand mean centered). In Model 1, regressed General ETG disclosure on team satisfaction, which, explained a significant portion of variance, *R^2^* = .14, *p* = .008. We found a significant, positive effect of General ETG disclosure (B = .64, SE = .22, *p* = .006) on team satisfaction. In Model 2, we added in the two potential mediators of commitment and trust, which resulted in a large, significant change in *R^2^,* Δ*R^2^* = .20, *p* = .000. In this model, ETG disclosure retained a marginally significant positive effect, and there were marginally significant, positive effects of both commitment (B = .34, SE = .20, *p* =.098), and trust (B = .23, SE = .13, *p* = .080). We then performed a test of mediation for both potential mediators simultaneously, with general ETG disclosure as the predictor (while controlling for team size), using the PROCESS macro for SPSS (Hayes, 2012: model 4). We did not find evidence of mediation for the effects of ETG disclosure on Team Satisfaction for commitment, indirect effect = .19, SE = .14, 95% CI = [-.008, .63]. However, there was no significant mediation for trust, indirect effect = .06, SE = .07, 95% CI = [-.05, .27]).

**The Direct and Indirect Effects of Disclosing Different Types of ETGs**

So far, we have shown that ETG disclosure, in general, has positive effects on team performance, team viability, and team satisfaction, and that those effects are partially or fully mediated by perceptions of the target’s commitment to the team’s goal (negatively, in the case of team performance). In this next section, we explore whether disclosing different types of ETGs (Learning ETGs, Instrumental ETGs, and those that combine elements of both) have differential effects on our three outcomes of interest.. Like before, we used OLS regression in R controlling for team size (grand mean centered). To assess the indirect effects of different types of ETG disclosure, we included perceptions of target’s commitment and trust in the target in the model as mediators. These predictors were grand mean centered, in order to improve the interpretability of the coefficients (Hofmann & Gavin, 1998). We then used the PROCESS macro for SPSS (Hayes, 2012: Models 4 and 7), which uses bootstrapped confidence intervals to analyze the indirect effects of different types of ETG disclosure on team performance, team viability, and team satisfaction via perceptions of target’s commitment and trust in the target.

***Team Performance.*** Return to Table S2 for a summary of the results of the team performance regression models. In Model 3, we regressed Learning ETG disclosure and Instrumental ETG disclosure on Team Performance, controlling for team size, which jointly predicted team performance *R^2^,* = .07, *p* = .095. We found a positive effect of Learning ETG disclosure, (B = .74, SE = .32, *p* = .025), but no significant effect of Instrumental ETG disclosure (B = .29, SE = .33, *p* = .387). Next, we added an interaction term for Learning ETG disclosure and Instrumental ETG disclosure in Model 4, which did not result in a significant change in *R^2^,* Δ*R^2^* = -.02, n.s., and we found no significant interaction effect. In Model 5 we added commitment and trust to the model without the interaction term, which resulted in a small but non-significant change in *R^2^,* Δ*R^2^* = .05, n.s. We found a significant, negative effect of commitment (B = -.79, SE = .37, *p* = .037) but no significant effects for trust (B = .35, SE = .24, *p* = .147. We then performed a test of mediation both potential mediators simultaneously, with Learning ETG disclosure as the predictor (while controlling for team size), using the PROCESS macro for SPSS (Hayes, 2012: Model 4). Here, perceived commitment partially, and negatively, mediated the effects of Learning ETG disclosure on Team Performance, indirect effect = -.22, SE = .16, 95% CI = [-.68, -.004]. However, there was no significant mediation for trust, indirect effect = .03, SE = .09, 95% CI = [-.09, .31].

***Team Viability.*** Return to Table S3 for a summary of the results of the team viability regression models. In Model 3, we regressed Learning ETG disclosure and Instrumental ETG disclosure, which jointly predicted team viability *R^2^* = .07, *p* = .091 Here we found a marginally significant, positive effect of Learning ETG disclosure (B = .38, SE = .21, *p* = .081), and no significant effect of Instrumental ETG disclosure (B = .31, SE = .22, *p* = .161). Next, in Model 4, we added an interaction term for Learning ETG disclosure and Instrumental ETG disclosure, which resulted in small, marginally significant change in *R^2^,* Δ*R^2^* = .05, *p* = .067. In this Model, we found a significant main effect of Learning ETG disclosure (B = .74, SE = .28, *p* =.012), a significant main effect of Instrumental ETG disclosure (B = .71, SE = .30, *p* =.023), and a marginally significant interaction effect (B = -.76, SE = .40, *p* = .067), suggesting that ETGs that contain elements of both learning and instrumentality lead to marginally lower perceptions of team viability. In Model 5 we added commitment and trust to the model with the interaction term, which resulted in a large and significant change in *R^2^,* Δ*R^2^* = .25, *p* = .000. We found a marginally significant, positive effect of commitment (B = .41, SE = .21, *p* = .054) and for trust (B = .25, SE = .14, *p* = .071). We then performed a test of moderated mediation with both potential mediators simultaneously to test the degree to which perceptions of commitment and trust mediate the relationship between Learning ETG disclosure and team viability with and without elements of Instrumental ETG disclosure (while controlling for team size), using the PROCESS macro for SPSS (Hayes, 2012: Model 7). We find that perceived commitment mediated the effects of Learning ETG disclosure on team viability, but only when those Learning ETGs did not include elements of instrumentality (conditional indirect effect = .31, SE = .19, 95% CI = [.04, .79]). When Learning ETGs also contained elements of instrumentality, perceived commitment did not mediate the effects of disclosure on team viability (conditional indirect effect = .04, SE = .14, 95% CI = [-.21, .36]). There was no significant mediation for trust, neither for Learning ETGs without instrumentality (conditional indirect effect = .11, SE = .10, 95% CI = [-.02, .35]) or with instrumentality (conditional indirect effect = -.029, SE = .09, 95% CI = [-.28, .10]).

***Team Satisfaction.*** Return to Table S4 for a summary of the results of the team satisfaction regression models. In Model 3, we regressed Learning ETG disclosure and Instrumental ETG disclosure on Team Satisfaction, which predicted a marginally significant amount of variance, *R^2^* = .07, *p* = .050. We found a marginally significant, positive effect of Learning ETG disclosure (B = .35, SE = .20, *p* = .086), but no significant effect of Instrumental ETG disclosure (B = .34, SE = .21, *p* = .102). Next, we added an interaction term for Learning ETG disclosure and Instrumental ETG disclosure in Model 4, which did not result in a significant change in *R^2^,* Δ*R^2^* = .02, n.s. In this model, we found a significant main effect of Learning ETG disclosure (B = .60, SE = .27, *p* = .032), and a significant main effect of Instrumental ETG disclosure (B = .62, SE = .29, *p* = .038), but no interaction effect (B = -.52, SE = .39, *p* = .186. In Model 5 we added commitment and trust to the model without the interaction term, which resulted in a large and significant change in *R^2^,* Δ*R^2^* = .24, *p* = .000. Here Learning ETG disclosure and Instrumental ETG disclosure no longer retained their significant effects and we found a marginally significant, positive effect of commitment (B = .34, SE = .20, *p* = .098) and trust (B = .26, SE = .13, *p* =.060). We then performed a test of mediation with both potential mediators simultaneously, with Learning ETG disclosure as the predictor (while controlling for team size), using the PROCESS macro for SPSS (Hayes, 2012: Model 4). Here, we found no evidence of mediation of the effects of Learning ETG disclosure on Team Satisfaction via perceived commitment, indirect effect = .16, SE = .15, 95% CI = [-.07, .51] or via trust, indirect effect = .02, SE = .06, 95% CI = [-.08, .20].

**Table S1:** **Descriptive statistics, aggregation indices, and team level correlations**

| Variable | M | SD | ICC(1) | ICC(2) | R*_wg(j)_* | 1 | 2 |
| --- | --- | --- | --- | --- | --- | --- | --- |
| 1. ETG | - | - | - | - | - | - |  |
| 2. Learning | - | - | - | - | - | - | - |
| 3. Instrumental | - | - | - | - | - | - | - |
| 4. Team Size | 4.3 | 0.77 | - | - | - | 0.067 | -0.218 |
| 5. Commitment (of Target) | 4.3 | 0.77 | 0.13 | 0.32 | 0.95 | 0.392** | 0.302* |
| 6. Trust (in Target) | 6.05 | 0.92 | 0.3 | 0.58 | 0.94 | 0.116 | 0.110 |
| 7. Team Viability | 5.3 | 1.14 | 0.1 | 0.25 | 0.92 | 0.393** | 0.282* |
| 8. Team Satisfaction | 5.48 | 1.21 | 0.15 | 0.35 | 0.95 | 0.362** | 0.286* |
| 9. Team Performance | 13.37 | 1.16 | - | - | - | 0.299* | 0.330* |
|  |  |  |  |  |  |  |  |
| Variable | 3 | 4 | 5 | 6 | 7 | 8 | 9 |
| 1. ETG |  |  |  |  |  |  |  |
| 2. Learning |  |  |  |  |  |  |  |
| 3. Instrumental | - |  |  |  |  |  |  |
| 4. Team Size | 0.311* | - |  |  |  |  |  |
| 5. Commitment (of Target) | 0.147 | -0.126 | - |  |  |  |  |
| 6. Trust (in Target) | -0.096 | -0.220 | .634*** | - |  |  |  |
| 7. Team Viability | 0.154 | -0.150 | .596*** | .519*** | - |  |  |
| 8. Team Satisfaction | 0.169 | -0.191 | .551*** | .497*** | .923*** | - |  |
| 9. Team Performance | 0.112 | 0.072 | -.072 | 0.045 | 0.108 | 0.167 | - |

*Notes:* N = 52 teams. t *p* < .10; * *p* < .05; ** *p* < .01; *** *p* < .001 (two-tailed)

| **Table S2: Regressions - ETG disclosure on Team Performance** | | | | | |
| --- | --- | --- | --- | --- | --- |
|  | | | | | |
|  | *Dependent variable:* Team Performance | | | | |
|  | (1) | (2) | (3) | (4) | (5) |
| Team Size | -0.128 | -0.142 | -0.056 | -0.061 | -0.054 |
|  | (0.190) | (0.190) | (0.204) | (0.206) | (0.202) |
|  |  |  |  |  |  |
| ETG | 0.811^*^ | 1.159^**^ |  |  |  |
|  | (0.362) | (0.392) |  |  |  |
|  |  |  |  |  |  |
| Commitment |  | -0.785^*^ |  |  | -0.787^*^ |
|  |  | (0.371) |  |  | (0.367) |
|  |  |  |  |  |  |
| Trust |  | 0.301 |  |  | 0.354 |
|  |  | (0.237) |  |  | (0.240) |
|  |  |  |  |  |  |
| Learning ETG |  |  | 0.741^*^ | 0.892^*^ | 0.952^**^ |
|  |  |  | (0.320) | (0.440) | (0.328) |
|  |  |  |  |  |  |
| Instrumental ETG |  |  | 0.287 | 0.454 | 0.479 |
|  |  |  | (0.329) | (0.469) | (0.333) |
|  |  |  |  |  |  |
| Learning*Instrumental |  |  |  | -0.317 |  |
|  |  |  |  | (0.630) |  |
|  |  |  |  |  |  |
| Constant | 13.293^**^ | 13.087^**^ | 13.077^**^ | 13.018^**^ | 12.864^**^ |
|  | (0.835) | (0.834) | (0.881) | (0.896) | (0.876) |
|  |  |  |  |  |  |
|  | | | | | |
| Adjusted R^2^ | 0.061t | 0.106t | 0.068t | 0.053 | 0.116t |
| Model Comparison | - | 1-2 | - | 3-4 | 3-5 |
| ΔR^2^ | - | .05 | - | -.02 | .05 |
|  | | | | | |

*Notes:* N = 52; t *p*<0.1; ^*^*p*<0.05; ^**^*p*<0.01; continuous predictor variables grand mean centered

| **Table S3: Regressions – ETG disclosure on Team Viability** | | | | | |
| --- | --- | --- | --- | --- | --- |
|  | | | | | |
|  | *Dependent variable:* Team Viability | | | | |
|  | (1) | (2) | (3) | (4) | (5) |
| Team Size | -0.163 | -0.060 | -0.147 | -0.158 | -0.066 |
|  | (0.119) | (0.104) | (0.135) | (0.132) | (0.114) |
|  |  |  |  |  |  |
| ETG | 0.713^**^ | 0.422 |  |  |  |
|  | (0.228) | (0.214) |  |  |  |
|  |  |  |  |  |  |
| Commitment |  | 0.418^*^ |  |  | 0.414t |
|  |  | (0.203) |  |  | (0.209) |
|  |  |  |  |  |  |
| Trust |  | 0.244t |  |  | 0.249t |
|  |  | (0.130) |  |  | (0.135) |
|  |  |  |  |  |  |
| Learning ETG |  |  | 0.378t | 0.737^*^ | 0.392 |
|  |  |  | (0.212) | (0.281) | (0.257) |
|  |  |  |  |  |  |
| Instrumental ETG |  |  | 0.309 | 0.708^*^ | 0.431 |
|  |  |  | (0.217) | (0.300) | (0.269) |
|  |  |  |  |  |  |
| Learning*Instrumental |  |  |  | -0.756t | -0.371 |
|  |  |  |  | (0.403) | (0.353) |
|  |  |  |  |  |  |
| Constant | 5.651^**^ | 5.439^**^ | 5.770^**^ | 5.630^**^ | 5.462^**^ |
|  | (0.525) | (0.456) | (0.583) | (0.573) | (0.495) |
|  |  |  |  |  |  |
|  | | | | | |
| Adjusted R^2^ | 0.152^**^ | 0.389^**^ | 0.070t | 0.116^*^ | 0.363^**^ |
| Model Comparison | - | 1-2 | - | 3-4 | 4-5 |
| ΔR^2^ | - | .24^***^ | - | .05t | .25^***^ |
|  | | | | | |

*Notes:* N = 52; t *p*<0.1; ^*^*p*<0.05; ^**^*p*<0.01; continuous predictor variables grand mean centered

| **Table S4: Regressions - ETG disclosure on Team Satisfaction** | | | | | |
| --- | --- | --- | --- | --- | --- |
|  | | | | | |
|  | *Dependent variable:* Team Satisfaction | | | | |
|  | (1) | (2) | (3) | (4) | (5) |
| Team Size | -0.192 | -0.100 | -0.188 | -0.196 | -0.106 |
|  | (0.116) | (0.104) | (0.128) | (0.127) | (0.112) |
|  |  |  |  |  |  |
| ETG | 0.639^**^ | 0.392t |  |  |  |
|  | (0.220) | (0.215) |  |  |  |
|  |  |  |  |  |  |
| Commitment |  | 0.344t |  |  | 0.344t |
|  |  | (0.204) |  |  | (0.204) |
|  |  |  |  |  |  |
| Trust |  | 0.233t |  |  | 0.257t |
|  |  | (0.130) |  |  | (0.133) |
|  |  |  |  |  |  |
| Learning ETG |  |  | 0.353t | 0.602^*^ | 0.213 |
|  |  |  | (0.201) | (0.272) | (0.182) |
|  |  |  |  |  |  |
| Instrumental ETG |  |  | 0.344 | 0.620^*^ | 0.286 |
|  |  |  | (0.207) | (0.290) | (0.185) |
|  |  |  |  |  |  |
| Learning*Instrumental |  |  |  | -0.523 |  |
|  |  |  |  | (0.389) |  |
|  |  |  |  |  |  |
| Constant | 5.977^**^ | 5.777^**^ | 6.089^**^ | 5.992^**^ | 5.850^**^ |
|  | (0.508) | (0.458) | (0.554) | (0.554) | (0.486) |
|  |  |  |  |  |  |
|  | | | | | |
| Adjusted R^2^ | 0.144^**^ | 0.339^**^ | 0.095t | 0.110^*^ | 0.331^**^ |
| Model Comparison | - | 1-2 | - | 3-4 | 3-5 |
| ΔR^2^ | - | .20^***^ | - | .02 | .24^***^ |
|  | | | | | |

*Notes:* N = 52; t *p*<0.1; ^*^*p*<0.05; ^**^*p*<0.01; continuous predictor variables grand mean centered

**Supplementary Appendix B: Regression Tables without the team size covariate (includes all five experimental conditions)**

| **Table S5: Regressions - ETG disclosure on Team Performance** | | | | | | |
| --- | --- | --- | --- | --- | --- | --- |
|  | | | | | | |
|  | *Dependent variable:* Team Performance | | | | | |
|  |  | | | | | |
|  |  | | | | | |
|  | (1) | (2) | (3) | (4) | (5) | (6) |
|  | | | | | | |
| Positive Statement | 0.940^*^ | 1.536^**^ | 1.676^**^ | 1.459^**^ | 1.536^**^ | 1.548^**^ |
|  | (0.381) | (0.463) | (0.460) | (0.432) | (0.463) | (0.425) |
|  |  |  |  |  |  |  |
| ETG |  | 0.795^*^ | 1.133^**^ |  |  |  |
|  |  | (0.370) | (0.400) |  |  |  |
|  |  |  |  |  |  |  |
| Commitment |  |  | -0.775^*^ |  |  | -0.782^*^ |
|  |  |  | (0.370) |  |  | (0.365) |
|  |  |  |  |  |  |  |
| Trust |  |  | 0.315 |  |  | 0.344 |
|  |  |  | (0.230) |  |  | (0.233) |
|  |  |  |  |  |  |  |
| Learning ETG |  |  |  | 0.761^*^ | 0.909^*^ | 0.972^**^ |
|  |  |  |  | (0.319) | (0.446) | (0.330) |
|  |  |  |  |  |  |  |
| Instrumental ETG |  |  |  | 0.258 | 0.418 | 0.448 |
|  |  |  |  | (0.319) | (0.463) | (0.326) |
|  |  |  |  |  |  |  |
| Learning*Instrumental |  |  |  |  | -0.308 |  |
|  |  |  |  |  | (0.643) |  |
|  |  |  |  |  |  |  |
| Constant | 13.365^**^ | 12.769^**^ | 12.529^**^ | 12.846^**^ | 12.769^**^ | 12.659^**^ |
|  | (0.165) | (0.321) | (0.336) | (0.276) | (0.321) | (0.284) |
|  |  |  |  |  |  |  |
|  | | | | | | |
| Adjusted R^2^ | 0.075^*^ | 0.125^**^ | 0.158^**^ | 0.135^**^ | 0.124^*^ | 0.171^**^ |
| Model Comparison | - | 1-2 | 2-3 | 1-4 | 4-5 | 4-6 |
| ΔR^2^ | - | .05^*^ | .03 | .06^*^ | -.01 | .04 |
|  | | | | | | |

*Notes:* N = 64; t *p*<0.1; ^*^*p*<0.05; ^**^*p*<0.01; continuous predictor variables grand mean centered

| **Table S6: Regressions - ETG disclosure on Team Viability** | | | | | | |
| --- | --- | --- | --- | --- | --- | --- |
|  | | | | | | |
|  | *Dependent variable:* Team Viability | | | | | |
|  |  | | | | | |
|  |  | | | | | |
|  | (1) | (2) | (3) | (4) | (5) | (6) |
|  | | | | | | |
| Positive Statement | 0.037 | 0.556t | 0.351 | 0.373 | 0.556t | 0.351 |
|  | (0.251) | (0.301) | (0.249) | (0.290) | (0.305) | (0.253) |
|  |  |  |  |  |  |  |
| ETG |  | 0.692^**^ | 0.356 |  |  |  |
|  |  | (0.246) | (0.219) |  |  |  |
|  |  |  |  |  |  |  |
| Commitment |  |  | 0.514^*^ |  |  | 0.509^*^ |
|  |  |  | (0.202) |  |  | (0.207) |
|  |  |  |  |  |  |  |
| Trust |  |  | 0.278^*^ |  |  | 0.284^*^ |
|  |  |  | (0.126) |  |  | (0.131) |
|  |  |  |  |  |  |  |
| Learning ETG |  |  |  | 0.431t | 0.783^*^ | 0.337 |
|  |  |  |  | (0.219) | (0.300) | (0.262) |
|  |  |  |  |  |  |  |
| Instrumental ETG |  |  |  | 0.234 | 0.614t | 0.344 |
|  |  |  |  | (0.219) | (0.312) | (0.266) |
|  |  |  |  |  |  |  |
| Learning*Instrumental |  |  |  |  | -0.733t | -0.290 |
|  |  |  |  |  | (0.433) | (0.361) |
|  |  |  |  |  |  |  |
| Constant | 5.504^**^ | 4.985^**^ | 5.229^**^ | 5.168^**^ | 4.985^**^ | 5.228^**^ |
|  | (0.112) | (0.213) | (0.184) | (0.190) | (0.216) | (0.188) |
|  |  |  |  |  |  |  |
|  | | | | | | |
| Adjusted R^2^ | -0.016 | 0.085^*^ | 0.397^**^ | 0.031 | 0.059 | 0.377^**^ |
| Model Comparison | - | 1-2 | 2-3 | 1-4 | 4-5 | 4-6 |
| ΔR^2^ | - | .10^**^ | .31^***^ | .05t | .03t | .32^***^ |
|  | | | | | | |

*Notes:* N = 65; t *p*<0.1; ^*^*p*<0.05; ^**^*p*<0.01; continuous predictor variables grand mean centered

| **Table S7: Regressions - ETG disclosure on Team Satisfaction** | | | | | | |
| --- | --- | --- | --- | --- | --- | --- |
|  | | | | | | |
|  | *Dependent variable:* Team Satisfaction | | | | | |
|  |  | | | | | |
|  |  | | | | | |
|  | (1) | (2) | (3) | (4) | (5) | (6) |
|  | | | | | | |
| Positive Statement | 0.135 | 0.596^*^ | 0.427t | 0.473t | 0.596^*^ | 0.398t |
|  | (0.224) | (0.270) | (0.227) | (0.257) | (0.273) | (0.211) |
|  |  |  |  |  |  |  |
| ETG |  | 0.615^**^ | 0.343t |  |  |  |
|  |  | (0.220) | (0.200) |  |  |  |
|  |  |  |  |  |  |  |
| Commitment |  |  | 0.396^*^ |  |  | 0.388^*^ |
|  |  |  | (0.185) |  |  | (0.184) |
|  |  |  |  |  |  |  |
| Trust |  |  | 0.271^*^ |  |  | 0.290^*^ |
|  |  |  | (0.115) |  |  | (0.118) |
|  |  |  |  |  |  |  |
| Learning ETG |  |  |  | 0.421^*^ | 0.659^*^ | 0.229 |
|  |  |  |  | (0.194) | (0.268) | (0.167) |
|  |  |  |  |  |  |  |
| Instrumental ETG |  |  |  | 0.248 | 0.504t | 0.229 |
|  |  |  |  | (0.194) | (0.279) | (0.165) |
|  |  |  |  |  |  |  |
| Learning*Instrumental |  |  |  |  | -0.495 |  |
|  |  |  |  |  | (0.387) |  |
|  |  |  |  |  |  |  |
| Constant | 5.653^**^ | 5.192^**^ | 5.390^**^ | 5.316^**^ | 5.192^**^ | 5.418^**^ |
|  | (0.100) | (0.191) | (0.168) | (0.168) | (0.193) | (0.144) |
|  |  |  |  |  |  |  |
|  | | | | | | |
| Adjusted R^2^ | -0.010 | 0.088^*^ | 0.376^**^ | 0.055t | 0.065t | 0.372^**^ |
| Model Comparison | - | 1-2 | 2-3 | 1-4 | 4-5 | 4-6 |
| ΔR^2^ | - | .10^**^ | .29^***^ | .07t | .01 | .31^***^ |
|  | | | | | | |

*Notes:* N = 64; t *p*<0.1; ^*^*p*<0.05; ^**^*p*<0.01; continuous predictor variables grand mean centered
